# *Escherichia coli* Maltose-Binding Protein Induces M1 Polarity of RAW264.7 Macrophage Cells via a TLR2- and TLR4-Dependent Manner

**DOI:** 10.3390/ijms16059896

**Published:** 2015-04-30

**Authors:** Wan Wang, Hong-Yan Yuan, Guo-Mu Liu, Wei-Hua Ni, Fang Wang, Gui-Xiang Tai

**Affiliations:** 1 Department of Immunology, College of Basic Medical Science, Jilin University, 126 Xinmin Street, Changchun 130021, China; E-Mails: wangwan0106@yahoo.com (W.W.); yuanhy@jlu.edu.cn (H.-Y.Y.); liuguomu@126.com (G.-M.L.); niwh5566@jlu.edu.cn (W.-H.N.); fangfang5460@126.com (F.W.); 2 Department of Breast Surgery, China-Japan Union Hospital, Jilin University, 126 Xiantai Blvd, Changchun 130033, China

**Keywords:** maltose-binding protein, classically activated macrophages, toll-like receptor 2, toll-like receptor 4

## Abstract

Maltose-binding protein (MBP) is a critical player of the maltose/maltodextrin transport system in *Escherichia coli*. Our previous studies have revealed that MBP nonspecifically induces T helper type 1 (Th1) cell activation and activates peritoneal macrophages obtained from mouse. In the present study, we reported a direct stimulatory effect of MBP on RAW264.7 cells, a murine macrophage cell line. When stimulated with MBP, the production of nitric oxide (NO), IL-1β, IL-6 and IL-12p70, and the expressions of CD80, MHC class II and inducible nitric oxide synthase (iNOS) were all increased in RAW264.7 cells, indicating the activation and polarization of RAW264.7 cells into M1 macrophages induced by MBP. Further study showed that MBP stimulation upregulated the expression of TLR2 and TLR4 on RAW264.7 cells, which was accompanied by subsequent phosphorylation of IκB-α and p38 MAPK. Pretreatment with anti-TLR2 or anti-TLR4 antibodies largely inhibited the phosphorylation of IκB-α and p38 MAPK, and greatly reduced MBP-induced NO and IL-12p70 production, suggesting that the MBP-induced macrophage activation and polarization were mediated by TLR2 and TLR4 signaling pathways. The observed results were independent of lipopolysaccharide contamination. Our study provides a new insight into a mechanism by which MBP enhances immune responses and warrants the potential application of MBP as an immune adjuvant in immune therapies.

## 1. Introduction

Maltose-binding protein (MBP) is a high-affinity maltose/maltodextrin-binding protein encoded by *malE* gene, and functions in capture and transportation of maltodextrins in *Escherichia coli* (*E. coli*) [1]. When fused with a recombinant protein, MBP can increase the solubility of the fusion protein by a poorly understood mechanism, and thus is commonly used to improve the yield, facilitate the purification and enhance the stability of fusion proteins [2,3]. Recently, MBP has been used as a chaperone component in various vaccines against pathogenic bacteria and viruses to enhance the immunogenicity of recombinant protein-MBP fusion protein vaccines [4,5,6]. MBP has been shown to induce dendritic cell (DC) activation and production of proinflammatory cytokines [7]. One of our previous studies has shown that MBP immunization induces activation of T helper type 1 (Th1) and natural killer (NK) cells in a mouse lung carcinoma model [8]. Furthermore, combined immunization with MBP and Bacillus Calmette-Guerin vaccine enhances the activation of macrophages *in vivo* [8]. The immunoadjuvant effect of MBP has been further demonstrated in our follow-up study of mouse peritoneal macrophages stimulated with lipopolysaccharide (LPS), and the activity of MBP is likely to be mediated by toll-like receptor 2 (TLR2) and TLR4 signaling pathways [9]. These findings suggest that MBP itself possesses potent immune enhancing properties. To gain a better insight into the mechanism of adjuvanticity by which MBP activates multiple immune cells including Th1 cells, macrophages and NK cells, further investigation is largely needed.

Macrophages are complex immune cells and play critical roles in innate and adaptive immune responses. They can be classified into M1 and M2 subsets based on the activation stimuli, function, and cytokine production. M1 macrophages, activated by LPS or IFN-γ, express a spectrum of proinflammatory cytokines, chemokines and effector molecules, such as IL-1β, IL-6, IL-12 and inducible nitric oxide synthase (iNOS); M2 macrophages, activated by IL-4, express a wide array of anti-inflammatory molecules, such as IL-10, TGF-β and arginase-1 (Arg-1). Our previous studies have shown that MBP enhances the production of inflammatory mediators and nitric oxide (NO) in mouse peritoneal macrophages and in macrophage cell lines [8,9,10]. Therefore, we hypothesize that, as a potent proinflammatory stimulus, MBP has the ability to polarize macrophages into M1 lineage.

In the present study, we investigated the effect of MBP on activation and polarization of murine macrophage RAW264.7 cells. Expression of markers for macrophage activation including CD80, MHC class I and class II was analyzed, as well as the pinocytosis of RAW264.7 cells induced by MBP. Simultaneously, production of NO, IL-1β, IL-6, IL-12p70, and expression of iNOS, which have been identified as specific markers for polarized M1 macrophages, were analyzed. To further explore the underlying mechanism, expression of TLR2 and TLR4, and phosphorylation of signaling molecules involved in the nuclear factor-κB (NF-κB) and p38 MAPK pathways were profiled in RAW264.7 cells with MBP stimulation.

## 2. Results

### 2.1. Maltose-Binding Protein (MBP) Enhances Nitric Oxide (NO) and Inflammatory Cytokine Secretion in RAW264.7 Cells

**Figure 1:**
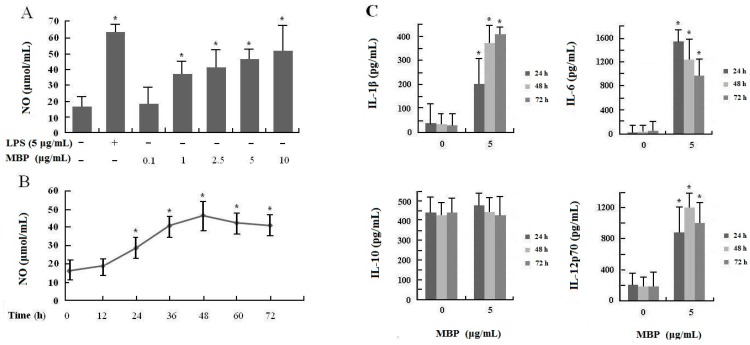
Effects of MBP on NO production and cytokine secretion in RAW264.7 macrophage cells. (**A**) RAW264.7 cells were treated with MBP (0.1–10 μg/mL) or LPS (5 μg/mL) for 48 h; (**B**) RAW264.7 cells were treated with 5 μg/mL MBP for 12–72 h. NO production was measured by the Griess reaction; (**C**) RAW264.7 cells were treated with 5 μg/mL MBP or left untreated. The culture supernatants were collected after 24, 48, or 72 h and examined by ELISA for cytokine secretion. Results were presented as mean ± SD of three independent experiments, each performed in triplicate. * *p* < 0.05 compared with the untreated control.

NO has been identified as one of the major effector molecules produced by activated macrophages, and is the main catabolite of iNOS in M1 macrophages [11,12,13]. To explore the effect of MBP on production of NO in RAW264.7 macrophage cells, we examined the NO levels in the culture supernatants of cells stimulated with various concentrations of MBP (0.1–10 μg/mL) for 48 h and those with 5 μg/mL MBP for 12 to 72 h. RAW264.7 cells stimulated with LPS (5 μg/mL) were used as positive control. The results showed that MBP significantly increased NO production in RAW264.7 cells in a dose and time-dependent manner (Figure 1A,B), suggesting that MBP induced activation and potentiates M1 polarity of RAW264.7 macrophage cells.

To investigate the possible role of MBP as an inflammation stimulus, effects of MBP on induction of proinflammatory cytokines, such as IL-1β, IL-6 and IL-12p70, and anti-inflammatory cytokine IL-10 in RAW264.7 cells, were examined. As shown in Figure 1C, MBP significantly induced IL-1β, IL-6 and IL-12p70 production in RAW264.7 cells compared to untreated controls (*p* < 0.01), but had no effect on IL-10 production. The results suggested that MBP promoted polarization of RAW264.7 cells into M1 lineage by increasing the production of M1 specific proinflammatory cytokines.

### 2.2. MBP Promotes Pinocytic Activities of RAW264.7 Cells with no Effect on Cell Viability

**Figure 2:**
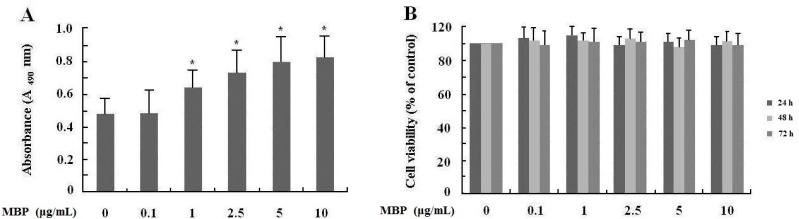
Effects of MBP on pinocytosis and viability of RAW264.7 macrophage cells. (**A**) RAW264.7 cells were treated with MBP (0.1–10 μg/mL) for 24 h. Pinocytosis was evaluated after incubating with neutral red dye for 1 h; (**B**) RAW264.7 cells were treated with MBP at different concentrations for 24, 48 or 72 h. Cell viability was determined by WST assay, and absorbance was measured at 450 nm. Results were presented as mean ± SD of three independent experiments, each performed in triplicate. * *p* < 0.05 compared with the untreated control.

Pinocytic activity is one of the most important functions of macrophages in innate immune response [13,14]. To assess the effects of MBP on macrophage functions, the pinocytic activities of RAW264.7 cells were evaluated by uptake of neutral red, a eurhodin dye that could be engulfed by activated macrophages, and the absorbance of cell lysates correlated with the pinocytic activity of cells. As shown in Figure 2A, MBP remarkably promoted the pinocytic activities of RAW264.7 cells, which further indicated the MBP-induced M1 polarity in RAW264.7 cells.

Furthermore, we examined the effect of MBP on viability of RAW264.7 cells. The results showed that there was no significant difference in the viability of RAW264.7 cells treated with various concentrations of MBP (Figure 2B), indicating that MBP did not affect the viability of treated cells.

### 2.3. MBP Upregulates Expression of Markers for Macrophage Activation and M1 Polarization in RAW264.7 Cells

**Figure 3:**
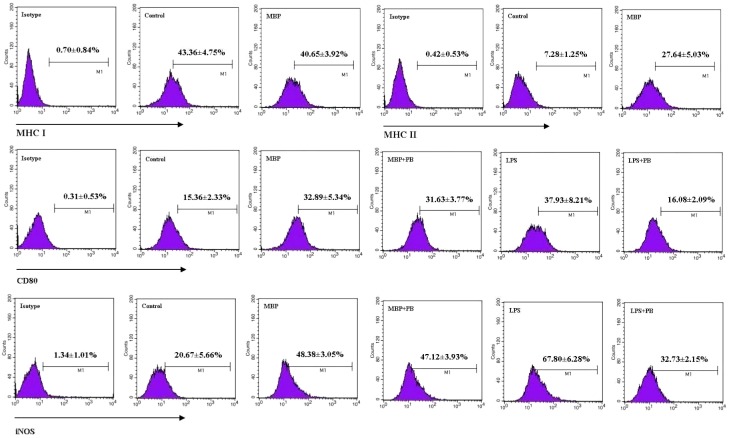
Effects of MBP on expression of MHC class I, MHC class II, CD80 and iNOS in RAW264.7 macrophages. RAW264.7 cells were treated with 5 μg/mL of MBP or 1 μg/mL of LPS for 48 h in the presence or absence of 5 μg/mL of polymyxin B (PB). Expression of MHC class I, class II, CD80 and iNOS was analyzed by flow cytometry using specific antibodies and isotype controls. Percentages of cells expressing each of these molecules were calculated. Representative flow plots are shown, and results from five independent experiments are presented as mean ± SD.

MHC class I and class II molecules are the important surface molecules of macrophage, and involved in presentation of endogenous and exogenous antigens, respectively [13,15]. To explore possible effect of MBP on presentation of antigens, expression of MHC class I and MHC class II on the surface of RAW264.7 cells treated with MBP were examined by flow cytometry. The results showed that MBP significantly upregulated the expression of MHC class II molecule on the surface of RAW264.7 cells, whereas MHC class I expression was not altered (Figure 3, upper panel).

To confirm the role of MBP in macrophage activation, we examined the expression of CD80, a classical surface marker of activated macrophages. To further eliminate the effect from potential LPS contamination in the MBP preparation, RAW264.7 cells were stimulated with MBP or LPS in the presence or absence of the LPS-binding antibiotic polymyxin B. We found that CD80 expression increased markedly on RAW264.7 cell surface after treatment with MBP (Figure 3, middle panel). Addition of polymyxin B did not influence MBP-induced upregulation of CD80, but as expected, greatly inhibited the expression of CD80 induced by LPS at 1 μg/mL (the equivalent of up to 10,000 endotoxin units/mL). These results suggested that MBP directly promoted the activation of RAW264.7 cells.

To provide additional evidence on MBP-induced M1 polarization, we examined the expression of iNOS, an important marker for M1 macrophages that functions to catabolize l-arginine to NO and citrulline [11,15]. Polymyxin B was added as previously described. Significantly higher iNOS expression was observed in cells treated with MBP compared with untreated controls (Figure 3, lower panel), which was consistent with increased NO production as shown in Figure 1. Similarly, addition of polymyxin B did not affect upregulation of iNOS in MBP-treated RAW264.7 cells while inhibited its expression in LPS-stimulated cells, indicating that the effect from endotoxin contamination in our MBP preparation was minimal and would not be a confounding factor in our analysis. These results further suggested that MBP polarized RAW264.7 cells into M1 macrophages.

### 2.4. MBP Activates TLR2 and TLR4 Expressions on RAW264.7 Cells

**Figure 4:**
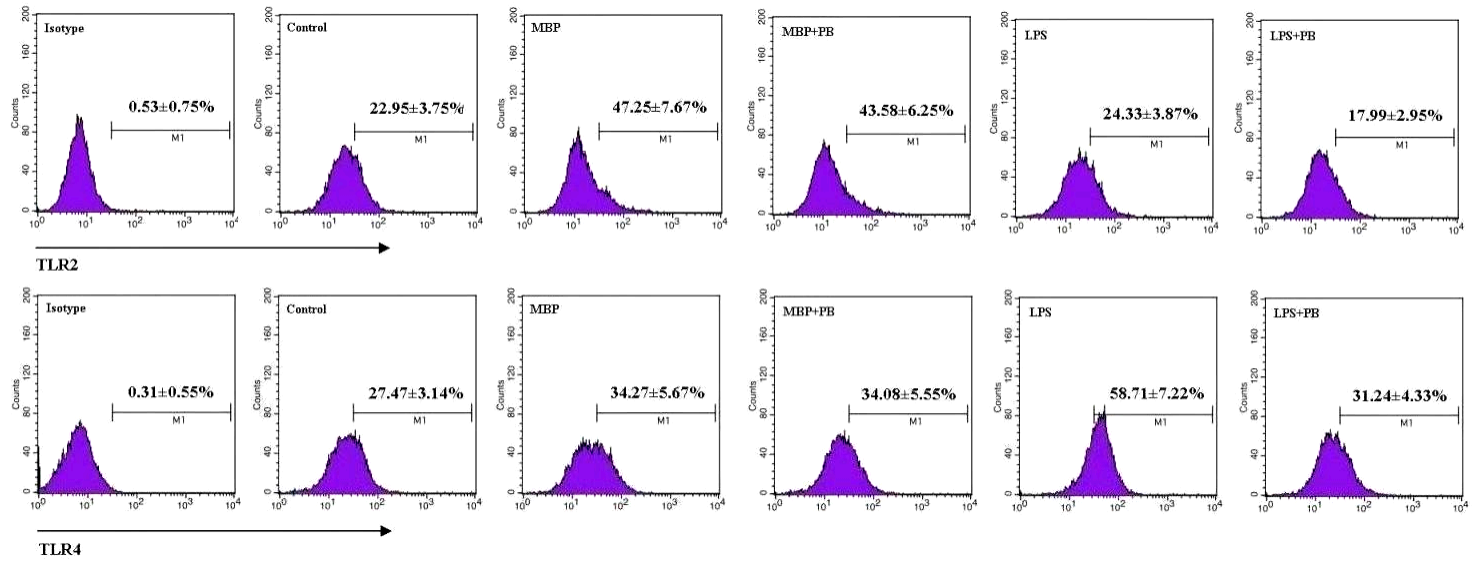
Effects of MBP on expression of TLR2 and TLR4 in RAW264.7 macrophages. RAW264.7 cells were treated with 5 μg/mL of MBP or 1 μg/mL of LPS for 48 h in the presence or absence of 5 μg/mL of polymyxin B (PB). Expression of TLR2 and TLR4 was analyzed by flow cytometry. Representative flow plots are shown, and results from three independent experiments are presented as mean ± SD.

Previous studies in our laboratory and others have demonstrated that MBP activates signaling transduction for DC maturation via TLR4 [7], enhances the viability of U937 monocytic cells through a TLR2-mediated pathway [10], and potentiates M1 polarity in mouse peritoneal macrophages in TLR2/4-dependent manner [9]. To investigate whether the effects of MBP on RAW264.7 cells were also mediated through TLR2 and/or TLR4 pathways, we first examined the cell surface expression of TLR2 and TLR4 in MBP-treated RAW264.7 cells. Polymyxin B was added as previously described to control for potential endotoxin contamination. We found that both TLR2 and TLR4 were upregulated when RAW264.7 cells were treated with MBP, which was not affected by addition of polymyxin B (Figure 4). However, LPS-induced TLR4 expression was abrogated by polymyxin B. These results indicated that MBP may promote the activation and polarization of RAW264.7 cells via TLR2 and TLR4 pathways.

### 2.5. MBP Activates NF-κB and p38 MAPK Signaling Pathways via TLR2 and TLR4 to Induce M1 Polarity

**Figure 5:**
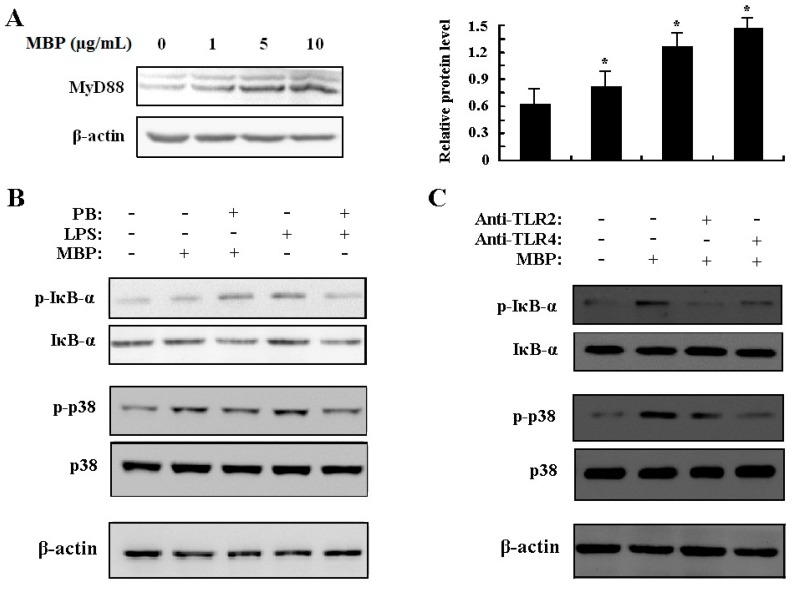
Effects of MBP on activation of NF-κB and p38 MAPK via TLR2 and TLR4. (**A**) RAW264.7 cells were treated with MBP (1, 5, 10 μg/mL) for 6 h. MyD88 expression was determined by Western blotting using whole cell lysates. MyD88 protein levels relative to the endogenous control β-actin were quantified and presented in bar graph. * *p* < 0.05 compared with the untreated control; (**B**) RAW264.7 cells were treated with MBP (1 μg/mL) or LPS (1 μg/mL) for 6 h in the presence or absence of 5 μg/mL polymyxin B; (**C**) RAW264.7 cells were pretreated with 20 μg/mL anti-TLR2 or anti-TLR4 antibodies prior to addition of 1 μg/mL MBP for 60 min. IκB-α, p38 MAPK and the phosphorylated proteins were detected by Western blotting using whole cell lysates.

MyD88 is an important adaptor protein that is recruited to all TLRs with the exception of TLR3 in response to TLR ligand engagement to mediate inflammatory responses to microbial components [16,17,18]. To examine the role of MyD88 in MBP-mediated response, expression of MyD88 was assessed by Western blotting. The results showed that MyD88 was upregulated in MBP-treated RAW264.7 cells (Figure 5A), which indicated the possible involvement of MyD88 in the process of MBP stimulation.

The cytokine secretion profile of MBP-stimulated RAW264.7 cells suggested that MBP functions as a proinflammatory agent and induces M1 polarization. To investigate the molecular mechanism that leads to this change, we measured the downstream IκB-α and p38 MAPK activation in MBP-treated RAW264.7 cells. Signaling through the NF-κB pathway has been associated with inflammatory events resulting in increased level of IκB-α phosphorylation. Resting unphosphorylated IκB-α interacts with the transcription factor NF-κB in the cytoplasm of cells. Phosphorylation of IκB-α results in release and consequent nuclear translocation of NF-κB. Treatment with 1 μg/mL MBP resulted in a significant increase in IκB-α and p38 MAPK phosphorylation with no effect on their total protein levels (Figure 5B). This result was not due to LPS contamination since polymyxin B treatment did not reverse the effect caused by our MBP preparation. In contrast, suppression of LPS-induced phosphorylation of IκB-α and p38 by pretreatment with polymyxin B was observed (Figure 5B).

To further confirm that MBP functions through TLR2 and TLR4 to activate downstream NF-κB and MAPK signaling pathways, TLR2 and TLR4 neutralizing antibodies were used to block the initiation of signal transduction. As shown in Figure 5C, pretreatment with anti-TLR2 or anti-TLR4 antibodies greatly inhibited the phosphorylation of IκB-α and p38 with no effect on total protein levels. These results, together with the previously shown upregulated expression of TLR2/4 in MBP-treated RAW264.7 cells, strongly suggested that both TLR2 and TLR4 mediate, at least partially, the activating and polarizing effects of MBP.

**Figure 6:**
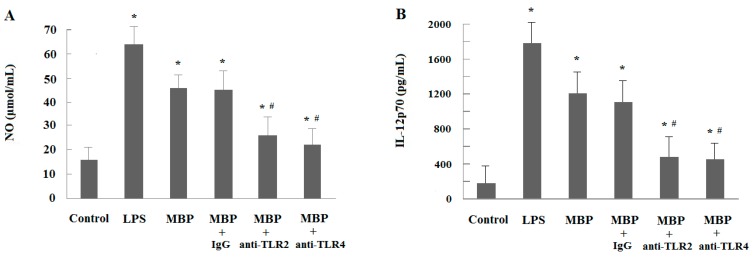
Effects of MBP-induced activation and polarization of RAW264.7 cells via TLR2 and TLR4. RAW264.7 cells were pre-incubated with anti-TLR2 or anti-TLR4 (20 μg/mL) antibody for 2 h prior to the addition of MBP or LPS (5 μg/mL) for 48 h. Mouse IgG2a (20 µg/mL) was used as isotype control. (**A**) MBP-induced NO production in RAW264.7 cells was partially inhibited by either anti-TLR2 or anti-TLR4; (**B**) MBP-induced M1 polarization of RAW264.7 cells was greatly inhibited by either anti-TLR2 or anti-TLR4. Results were presented as mean ± SD from three independent experiments, each performed in triplicate. * *p* < 0.05 compared with the untreated control. ^#^ *p* < 0.05 compared with the MBP-treated group.

A similar effect was detected when examining the NO production of MBP-treated RAW264.7 macrophages (Figure 6A). The MBP-induced NO production of macrophages was partially inhibited by either anti-TLR2 or anti-TLR4 (*p* < 0.05), while, the effect of mouse isotype control IgG on NO production was minimal. The results indicated that TLR2 and TLR4 are involved in the activation of RAW264.7 cells induced by MBP. To further confirm the involvement of TLR2 and TLR4 in the polarization of MBP-induced macrophages, IL-12p70 secretion was examined. Treatment with anti-TLR2 or anti-TLR4 partially attenuated the MBP-induced IL-12 enhancement, which indicated that MBP-induced M1 polarization of RAW264.7 cells can be partially inhibited by anti-TLR2 or anti-TLR4 (Figure 6B). These data suggest that TLR-2 and TLR4 are involved in activation and polarization of RAW264.7 cells mediated by MBP.

## 3. Discussion

MBP has been used as a chaperone component in vaccines to enhance antigen-specific humoral and cellular immune responses in immunized animals [2,3,4,5,6,7]. In addition to the immunostimulatory role, previous studies have also shown that MBP provides intrinsic maturation stimulus to DCs [7], induces Th1 cell activation, and increases the production of NO in mouse peritoneal macrophages [8,9]. In the present study, our results indicated that MBP had a direct effect on the activation and polarization of RAW264.7 macrophage cells into M1 lineage. Upon stimulation with MBP, increased NO production and elevated expression of CD80 and MHC class II molecules in RAW264.7 cells were observed, suggesting that MBP directly induced macrophage activation. Furthermore, an increased release of the typical M1 marker cytokines such as IL-1β, IL-6, and IL-12p70, and an elevated expression of iNOS were observed in MBP-treated RAW264.7 cells, suggesting that MBP polarized RAW264.7 cells into M1 macrophages.

The phenotypic changes detected in MBP-treated RAW264.7 cells were equivalent to those induced by LPS, a classical inducer of macrophage activation and polarization. Since MBP is a protein product of *E. coli*, the critical doubt about LPS contamination was a major concern in our study. To eliminate the effect of LPS that could potentially compromise our results, MBP was prepared through ultrafiltration techniques, and the residual endotoxin level in our MBP preparation did not exceed 0.06 EU/mL. To further assess the outcome from residual endotoxin contamination, polymyxin B was added to RAW264.7 cells in most of the experiments and the results were compared to those without polymyxin B treatment. Polymyxin B, a cyclic cationic polypeptide antibiotic produced by the soil bacterium *Paenibacillus polymixa*, specifically blocks the biological effects of LPS through binding to lipid A, the toxic component of LPS [19]. Our results showed a minimal effect of polymyxin B when used with MBP, indicating that the possibility of LPS contamination could be ruled out.

We further investigated the possible mechanisms by which MBP activated and polarized RAW264.7 macrophage cells. Previous studies have indicated critical roles for TLR2 and TLR4 signaling pathways in MBP-induced immune cell activation [7,9,10]. Moreover, MBP is a bacterial product, while TLR2 and TLR4 are well known for their involvement in recognition of microbial cell wall components and initiation of antimicrobial immune responses [20]. Therefore, we hypothesized that TLR2 and TLR4 might be important receptors for MBP. In the present study, our results indicated that the activation and M1 polarization of RAW264.7 cells were accompanied by upregulation of the cell surface expressions of TLR2 and TLR4. In order to confirm that MBP act as a ligand for the pattern recognition receptors TLR2 and TLR4, which play important roles in the process of macrophage activation, polarization and functional exertion, we further studied the downstream signaling pathways. According to our results, IκB-α and p38 MAPK were both responsive to MBP stimulation by increasing the levels of IκB-α and p38 phosphorylation in RAW264.7 cells, suggesting that the signaling through NF-κB and p38 MAPK pathways were associated with macrophage activation and polarization. Furthermore, IκB-α and p38 phosphorylation was largely abrogated by pretreatment with anti-TLR4 or anti-TLR2 antibodies. Previous investigations have indicated that the adaptor protein MyD88 mediates the TLR signaling in response to stimulation [21,22]. In our study, increased MyD88 protein level upon MBP treatment was observed. Together, these results elucidated the regulatory network of MBP, in which MBP functioned through both TLR2 and TLR4 to activate the downstream NF-κB and p38 MAPK signaling pathways mediated at least partially by MyD88, and consequently resulted in macrophage activation, proinflammatory cytokine secretion, and M1 polarityin RAW264.7 macrophage cells. However, it remains unclear how MBP interacts with TLR2 and TLR4, and whether other adaptor molecules contribute to this process.

In summary, our study provides strong evidence that MBP, which has hitherto been considered as a potent proinflammatory stimulus in various immune cell types, can activate macrophage and polarize them into M1 lineage via TLR2 and TLR4 and the subsequent activation of NF-κB and p38 MAPK signaling pathways. MBP may have intrinsic adjuvant-like properties to enhance immune responses. MBP could serve as a strong candidate in the development of effective immunoadjuvants to be used in vaccines and tumor immunotherapy.

## 4. Experimental Section

### 4.1. Reagents and Antibodies

MBP was obtained from an *E. coli* strain that carries the MBP expression vector pMAL-c2 (New England Biolabs, Hitchin, UK). Endotoxin was removed by first passing the MBP preparation through a polymyxin B-agarose column (Sigma-Aldrich, Saint Louis, MO, USA), followed by ultrafiltration techniques with Amicon Ultra-15 Centrifugal Filter Units plus Ultracel-10 (Merck Millipore, Billerica, MA, USA). MBP protein was subsequently tested for endotoxin remnants using a limulus amebocyte lysate-based assay (BioWhittaker, Atlanta, GA, USA). Endotoxin levels in the final preparation did not exceed 0.06 endotoxin units (EU)/mL in all tested samples. LPS (*E. coli* 055:B5) and polymyxin B sulfate salt were purchased from Sigma-Aldrich. Mouse IL-1β, IL-6, IL-10 and IL-12p70 enzyme-linked immunosorbent assay (ELISA) kits were purchased from eBiosciences (San Diego, CA, USA). Fluorescence conjugated antibodies specific for CD80, MHC class I, MHC class II, TLR2 and TLR4/MD-2 complex were obtained from BioLegend (San Diego, CA, USA). Functional grade purified antibodies against TLR2, TLR4/MD-2 and CD16/32 were obtained from eBiosciences. Antibodies specific for MyD88, iNOS and β-actin were purchased from Abcam (Cambridge, UK). Antibodies specific for IκB-α and p38 MAPK, and those specific for phosphorylated IκB-α and p38 were purchased from Cell Signaling Technology (Beverly, MA, USA). Horseradish peroxidase conjugated secondary antibodies were obtained from Santa Cruz Biotechnology (Santa Cruz, CA, USA).

### 4.2. Cell Culture

RAW264.7 macrophage cells were obtained from the American Type Culture Collection (ATCC; Rockville, MD, USA) and cultured in RPMI-1640 medium containing 10% fetal bovine serum (FBS; both from Invitrogen, Carlsbad, CA, USA), 100 U/mL penicillin, and 100 μg/mL streptomycin (Gibco BRL, Gaithersburg, MD, USA) in a humidified 5% CO_2_ 37 °C incubator. For NO production, cytokine secretion, cell viability and pinocytosis assays, RAW264.7 cells were seeded in triplicate in 96-well plates at 1 × 10^5^ cells/well and treated with various concentrations of MBP (0.1–10 μg/mL) or 5 μg/mL of LPS for certain hours as specified in figure legends. For flow cytometry and Western blot analysis, cells were treated with MBP or LPS (1 or 5 μg/mL) in the presence or absence of 5 μg/mL polymyxin B prior to being seeded in 24-well plates at 4 × 10^5^ cells/well for 48 or 6 h, respectively.

### 4.3. NO Production

As nitrite being a major stable product of NO, the concentration of NO in culture supernatant was determined by measuring the nitrite level using the Griess reagent (Sigma-Aldrich). Briefly, equal volumes of culture supernatant and Griess reagent (100 μL) were mixed for 10 min at room temperature. Nitrite levels were determined using a microplate reader (BioTek Instruments Inc., Winooski, VT, USA) at a wavelength of 540 nm. Sodium nitrite was used as a standard.

### 4.4. Cytokine Assay

Levels of IL-1β, IL-6, IL-10 and IL-12p70 in the supernatants of cultured cells were measured using commercial mouse IL-1β, IL-6, IL-10 and IL-12p70 ELISA kits according to the manufacturer’s protocols, respectively. The concentrations were expressed in pg/mL, and calculated from calibration curves from serial dilutions of murine recombinant standards in each assay.

### 4.5. Pinocytosis Assay

RAW264.7 cells were incubated in RPMI 1640 medium with 10% FBS in the presence or absence of MBP (0.1–10 μg/mL) at 37 °C for 24 h. Culture media were removed and 200 μL/well of 0.7% neutral red dye was added. Media were discarded after incubation for 1 h. The cells were washed twice with phosphate-buffered saline (PBS, pH 7.4, 0.01 mol/L) and then lysed in 200 μL of lysis solution (1:1 of 0.1 mol/L acetic acid and 100% ethanol) at 4 °C overnight. Absorbance was measured at 490 nm.

### 4.6. Cell Viability Assay

Cell viability was assessed using a Cell Counting kit with water-soluble tetrazolium (WST) according to the manufacturer’s instruction (Dojindo Molecular Technologies, Tokyo, Japan). Briefly, RAW264.7 cells were seeded in 96-well plates and incubated in 200 μL of RPMI 1640 medium with 10% FBS in the presence or absence of MBP (0.1–10 μg/mL) at 37 °C. WST reagent was added at 24, 48, or 72 h, and incubation was continued for an additional 1–2 h. Absorbance was detected at 450 nm.

### 4.7. Flow Cytometric Analysis

Expressions of MHC class I, MHC class II, CD80, iNOS, TLR2 and TLR4 in RAW264.7 cells were analyzed by flow cytometry. Briefly, RAW264.7 cells were washed in PBS supplemented with 1% FBS and 0.01% NaN_3_. Cells were incubated with CD16/32 at 4 °C for 30 min to block Fc-receptors, followed by incubation with fluorescence conjugated antibodies or isotype-matched control for 30 min on ice avoiding from light. Following the final washing step, labeled cells were analyzed by flow cytometry with FACSCalibur (BD Biosciences, Franklin Lakes, NJ, USA). The data were collected and analyzed with CellQuest software (BD Biosciences) to assess the percentage of fluorescence positive cells.

### 4.8. Blocking of TLR2/4

For TLR2 and TLR4 blocking assays, RAW264.7 cells were treated separately in different experiments. RAW264.7 cells were cultured in the presence of 20 µg/mL functional grade purified antibodies against mouse TLR2 or TLR4/MD-2 complex, and mouse IgG2a (20 µg/mL) (eBiosciences) was used as isotype control. After 2 h, 5 µg/mL MBP was added for an additional 48 h of culture. Then, NO production and IL-12p70 secretion were analyzed. LPS (5 µg/mL) was added as a control. For Western blot analysis, RAW264.7 cells were grown in 24-well plates and pretreated with 20 µg/mL functional grade purified anti-TLR2 or anti-TLR4 for 60 min, and then cells were stimulated with 1 µg/mL MBP or LPS in the presence or absence of 5 µg/mL polymyxin B (PB) for 6 h. After incubation for 6 h, cells were collected for Western blot analysis.

### 4.9. Western Blot Analysis

Cells were lysed in RIPA buffer (Thermo, Rockford, IL, USA) in the presence of the protease inhibitor leupeptin and the phosphatase inhibitor PhosSTOP (Roche, Indianapolis, IN, USA). Protein concentration was determined using the bicinchoninic acid (BCA) protein detection system (Bio-Rad, Hercules, CA, USA). Equal amounts of protein were separated by 10% SDS-PAGE and transferred to PVDF membranes (Millipore, Billerica, MA, USA). The membranes were blocked with 5% nonfat milk (GE Healthcare, Little Chalfont, UK) in Tris-buffered saline containing 0.1% Tween-20 (TBS-T) for 2 h at room temperature, and then incubated with primary antibodies against MyD88 (1:500), p38 MAPK and phospho-p38 MAPK (1:1000), IκB-α and phospho-IκB-α (1:1000) overnight at 4 °C, and β-actin was used as the endogenous control. After washing with TBS-T for three times, the membranes were incubated with secondary antibodies for 2 h at room temperature. Bands were visualized using SuperSignal chemiluminescence substrate from Pierce (Rockford, IL, USA) according to the manufacturer’s instructions.

### 4.10. Statistical Analysis

The measurement data were expressed as means ± standard deviation (SD). Differences between groups were compared using a one-way ANOVA followed by Student’s *t*-tests. A *p* value less than 0.05 (*p* < 0.05) was considered to be statistically significant. All statistical analyses were performed using SPSS 17.0 software (SPSS Inc., Chicago, IL, USA).

## 5. Conclusions

Our study provides strong evidence that MBP can activate macrophage and polarize them into M1 lineage via TLR2 and TLR4 and the subsequent activation of NF-κB and p38 MAPK signaling pathways. MBP may have intrinsic adjuvant-like properties to enhance immune responses. MBP could serve as a strong candidate in the development of effective immunoadjuvants to be used in vaccines and tumor immunotherapy.
